# Expression of transglutaminase 2 in human gut epithelial cells: Implications for coeliac disease

**DOI:** 10.1371/journal.pone.0287662

**Published:** 2023-06-27

**Authors:** Sunniva F. Amundsen, Jorunn Stamnaes, Knut E. A. Lundin, Ludvig M. Sollid

**Affiliations:** 1 KG Jebsen Coeliac Disease Research Centre, Institute of Clinical Medicine, University of Oslo, Oslo, Norway; 2 Department of Immunology, Oslo University Hospital—Rikshospitalet, Oslo, Norway; 3 Department of Gastroenterology, Oslo University Hospital—Rikshospitalet, Oslo, Norway; University Hospital of Bologna Sant’Orsola-Malpighi Polyclinic Department of Digestive System: Azienda Ospedaliero-Universitaria di Bologna Policlinico Sant’Orsola-Malpighi Dipartimento dell’apparato digerente, ITALY

## Abstract

**Background:**

Formation of complexes between transglutaminase 2 (TG2) and gluten can mechanistically explain why TG2 serves both as B-cell autoantigen and as an enzyme that creates deamidated gluten epitopes in coeliac disease (CeD). A model has been proposed where TG2 released from shed epithelial cells encounters high concentrations of dietary gluten peptides to form these TG2:gluten complexes. In this work we have characterised TG2 protein expression in gut epithelial cells in humans.

**Methods:**

Western blot analysis, immunofluorescence staining and mass spectrometry in combination with laser capture microdissection to gain spatial resolution were used to characterise TG2 expression in the epithelial cell layer of healthy and coeliac disease affected duodenum.

**Findings:**

TG2 is expressed in human duodenal epithelial cells, including cells in the apical region that are shed into the gut lumen. In untreated CeD the apical expression of TG2 is doubled. Enzymatically active TG2 is readily released from isolated human intestinal epithelial cells.

**Conclusion:**

Shed epithelial cells are a plausible source of pathogenic TG2 enzyme in CeD. Increased epithelial TG2 expression and increased epithelial shedding in active CeD may reinforce action of luminal TG2 in this condition.

## Introduction

Coeliac disease (CeD) is an autoimmune-like disorder of the small intestine caused by an inappropriate adaptive immune response towards dietary gluten peptides [1]. The endogenous enzyme TG2 plays an important role in the disease by converting neutral glutamine residues to negatively charged glutamate (deamidation). Deamidated gluten peptides bound to the disease-associated MHC class II molecules HLA-DQ2.5, HLA-DQ8 or HLA-DQ2.2 are recognised by pro-inflammatory CD4+ T cells that drive the subsequent inflammation in the small intestine [2, 3]. TG2 is also the target of disease-specific autoantibodies, which depend on intake of dietary gluten [4]. During deamidation, transient covalent complexes are formed between TG2 and gluten peptides. This complex formation led to the proposal of a hapten-carrier-like mechanism wherein TG2-specific B cells take up TG2:gluten complexes and present deamidated gluten peptides to gluten-specific CD4+ T cells [5].

In recent years, we have obtained experimental support for this model and demonstrated that preformed TG2:gluten complexes facilitate cross-talk between TG2-specific B cells and gluten-specific T cells both *in vitro* [6] and *in vivo* in mice [4]. However, if and where TG2:gluten complexes can form *in vivo* is still unclear. Surprisingly, as demonstrated in an experimental mouse model, TG2-specific B-cells show no sign of encountering TG2 antigen during their development [4] which suggests that little, if any, extracellular TG2 is present under normal physiological conditions. In addition, evidence indicates that the observations of extracellular TG2 in frozen tissue sections are largely artefacts, as cytosolic TG2 readily binds to extracellular matrix components during the tissue processing (Stamnaes *et al*. manuscript in preparation). Accordingly, TG2-mediated deamidation of gluten *in vivo* should be a rare event, which agrees poorly with the observation of rapid CD4+ T cell response to deamidated gluten upon oral gluten challenge of treated CeD patients [7, 8]. We recently proposed that the intestinal gut lumen represent a hitherto unappreciated location where TG2 and gluten are guaranteed to meet [9]. Intestinal epithelial cells are continuously shed into the gut lumen. Contrary to the previous belief that epithelial cells which are shed into the gut lumen are primarily apoptotic, newer findings support a notion that the majority of epithelial cells shed in the human jejunum are live with intact cytosolic enzymes [10]. Thus, TG2 released from shed epithelial cells will therefore encounter high concentrations of dietary gluten peptides, which could facilitate formation of TG2:gluten complexes. In support of this model, we have shown that TG2 released from isolated mouse small intestinal epithelium cells can form TG2:gluten peptide complexes that allow for uptake and crosstalk between TG2-specific B cells and gluten specific CD4+ T-cell *in vitro* [9].

To exert their function, TG2:gluten complexes must be taken up by immune cells in lymphoid tissue such as isolated lymphoid follicles or Peyer’s patches [11]. Two factors in particular will determine how much TG2:gluten complex is available for uptake; 1) the amount of TG2 enzyme that can be present in the gut lumen and 2) the stability of the TG2:gluten complex. We recently demonstrated that known immunogenic T-cell epitopes form stable complexes with TG2 that can be taken up by TG2-specific B cells [12]. In the study presented here, we have characterised the TG2 protein levels in intestinal epithelial cells from both healthy humans and patients with coeliac driven inflammation of the small intestine.

## Methods and materials

### Biological material

Duodenal biopsies were obtained by gastroduodenoscopy according to protocols approved by the Regional Ethics Committee of South-Eastern Norway (project 6544). The approved protocol conforms to the ethical guidelines of the 1975 Declaration of Helsinki. All participating subjects gave informed written consent. The diagnosis of CeD was made according to established guidelines and included positive serology (IgA antibodies to TG2 and/or IgG antibodies to deamidated gliadin peptides) and duodenal biopsies showing villous atrophy as well as other diagnostic facets of CeD (Marsh 3A-C) [13]. Biopsies were collected from untreated CeD (UCeD) patients that were on a gluten containing diet and treated CeD (TCeD) patients that had been on a gluten free diet for 1 year. For analysis of formalin fixed paraffin embedded (FFPE) tissue we also included duodenal biopsies of control subjects (CTR) who underwent gastroduodenoscopy for clinical complaints and who were not diagnosed with CeD. These biopsies had normal mucosal architecture with no sign of inflammation. Participants whose biopsies were used for EDTA-fraction analysis were recruited in 2020 or 2022. (TCeD, n = 11, 8/11 female, median age 42 years (range 22–61), UCeD n = 11, 7/11 female, median age 43 years (range 22–69). Biopsies used for laser capture microdissection (LCM) and immunofluorescence (IF) staining were collected between 2015–2018 and analysed in 2022 (total n = 11, 3/11 female, median age 27 years (range 23–87). The authors did not have access to information that could identify individual participants during or after data collection. An overview of patient biopsies used in this study is given in S1 Table. The animal work was approved by the Norwegian Food Safety Authority (Mattilsynet, Project ID 22198).

### Laser capture microdissection and sample work-up

Preparation and analysis using LCM and mass spectrometry (MS) was done as previously described [14]. Tissue sections of FFPE human duodenal biopsies or mouse small intestine (5 μm) were adhered to PEN-covered slides (Zeiss) and dried at 37°C. The dry sections were dewaxed in xylene and rehydrated using ethanol and water. Visualization of cells was done using Mayer’s haematoxylin solution (Sigma). A PALM MicroBeam laser capture microdissection system (Carl Zeiss MicroImaging, Munich, Germany) was used to collect the isolated tissue into 0.5 mL opaque adhesive cap tubes (Zeiss). For all samples about 350 000 μm^2^ of tissue was collected from either the apical or lateral part of the epithelial cell layer.

Dissected tissue was retrieved from the adhesive caps using 20 μl 0.1% ProteaseMax surfactant (Promega, WI, US) in 50 mM ammonium bicarbonate (ABC) and transferred to Low-Bind tubes (Eppendorf, Germany). The samples were heated for 90 min at 98°C followed by 1 h sonication in a water bath. Reduction was done by adding 2 μl 100 mM dithiothreitol and incubation at 56°C for 20 min before adding 2 μl 55 mM iodoacetamide for alkylation and incubating in 24°C for 30 min in the dark. Digestion was done by addition of 1.5 μl 0.01 μg/μl trypsin (Promega) with incubation overnight in 37°C and ended by acidification of the samples.

### Isolation of epithelial cells by EDTA-fractionation

Ten to twelve duodenal biopsies were collected in RPMI from each patient. The biopsies were transferred to 5 ml 2 mM EDTA/RPMI and incubated under rotation at 37°C for 10 min. Solid tissue was removed before the cells in suspension were spun down at 100 rcf at 4°C for 4 min. The cell pellet was dissolved in 1 ml PBS and spun down at 350 rcf for 7 min. The pellet was snap-frozen in liquid N_2_. The cells were stored in -70°C prior to use.

The EDTA fractions were thawed on ice and lysed using one of two alternative methods. The first method used Pierce RIPA-buffer (Thermo Scientific) containing 1x Halt Protease and Phosphatase inhibitor cocktail (Thermo Scientific). Cells suspended in RIPA-buffer was incubated on ice for 10 min with vortexing every 2–3 min, followed by sonication at 2x15 s at 25% amplitude. The samples were centrifuged at 13 000 rcf for 7 min at 4°C. For each replicate 10 μg of protein from the supernatant was precipitated on amine beads as described [15]. The second lysis method was via a freeze-thaw (FT) cycle, done to maintain TG2’s enzymatic activity, as previously described [9]. Here, the cell pellet was submerged in liquid N_2_ three times while being thawed at 37°C in between freezing. All quantification of lysate proteins was done using a BCA assay kit (Thermo Scientific). For digestion the proteins were dissolved in 50mM ammonium bicarbonate, reduced with dithiothreitol, alkylated with iodacetamide and digested with trypsin (1:50 enzyme:protein ratio; Promega) at 37°C overnight. Digested peptides were transferred to a new tube, acidified and the peptides were desalted by homemade C18 stage tips.

### Mass spectrometry analysis

The entire digested LCM-sample was injected onto the MS, whilst 200 ng protein of lysates from the EDTA-fraction was injected. Almost all MS experiments were performed on a TimsTOF fleX instrument (Bruker Daltonics, Bremen, Germany) coupled to a nanoElute nanoflow ultrahigh pressure liquid chromatography (LC) system (Bruker Daltonics), using a CaptiveSpray nanoelectrospray ion source (Bruker Daltonics). Peptide digest was loaded on a capillary C18 column (25 cm length, 75 μm inner diameter, 1.6 μm particle size, 120 Å pore size; IonOpticks, Fitzroy, VIC, AUS). Peptides were separated at 55°C using a 60 min gradient at a flow rate of 300 nl/min (mobile phase A (MPA): 0.1% formic acid (FA), mobile phase B (MPB): 0.1% FA in acetonitrile). A stepwise gradient of 2–17% MPB was applied for 26 mins, followed by a step from 17 to 25% MPB from 27 to 48 mins, 25–35% MPB from 48 to 61 mins, 35–85% MPB from 61 to 64 mins, and finished with a wash at 85% MPB for an additional 7 mins. The timsTOF fleX was operated in PASEF mode. Mass spectra for MS and MS/MS scans were recorded between m/z 100 and 1700. Ion mobility resolution was set to 0.60–1.60 V·s/cm over a ramp time of 100 ms. Data-dependent acquisition was performed using 10 PASEF MS/MS scans per cycle with a near 100% duty cycle. A polygon filter was applied in the m/z and ion mobility space to exclude low m/z, singly charged ions from PASEF precursor selection. An active exclusion time of 0.4 min was applied to precursors that reached 20 000 intensity units. Collisional energy was ramped stepwise as a function of ion mobility. Only the gluten-challenge MS data was not analysed using the aforementioned method, but with the method described in [14].

### Data analysis

Raw files from LC-MS/MS analyses were submitted to MaxQuant [16] (version 2.0.1.0) software for protein identification and label-free quantification. Parameters were set as follows: Carbamidomethyl (C) was set as a fixed modification and protein N-acetylation and methionine oxidation as variable modifications. First search error window of 20 ppm and mains search error of 6 ppm. Trypsin without proline restriction enzyme option was used, with two allowed mis-cleavages. Minimal unique peptides were set to one, and false discovery rate allowed was 0.01 (1%) for peptide and protein identification. The Uniprot database with human or mouse sequences was used. Generation of reversed sequences was selected to assign false discovery rate. Label-free quantification (LFQ) values were used to compare values between samples, iBAQ values were used to compare protein levels within samples. Relative levels of TG2 were calculated by dividing the TG2 rank number on the total number of detected proteins within one sample.

### Immunofluorescence staining and quantification

Three μm FFPE tissue sections were deparaffinised, subjected to heat-induced antigen retrieval (20 min at 98°C) and stained for TG2 protein using a custom polyclonal rabbit-anti-human TG2 antibody (Pacific immunology, CA, US) at 1μg/ml over night at 4°C followed by detection using donkey-anti-rabbit-IgG-Cy3 (Jackson ImmunoResearch). Nuclei were counterstained with 40,6-diamidino-2-phenylindole (DAPI) and slides were mounted with ProLong Diamond Antifade Mountant (ThermoFisher). Slides were imaged on an inverted Nikon fluorescence microscope (Nikon Eclipse Ti-S; Nikon, Tokyo, Japan) and images were processed in Fiji (ImageJ) [17]. Two-three images were captured from each patient biopsy (UCeD n = 3, TCeD, n = 2, CTR n = 3) using identical acquisition settings. Fluorescence intensity was measured in FIJI from unprocessed images acquired with identical microscope settings. Apical and lateral epithelial regions were defined using the freehand line tool and mean region signal intensity was measured. From each image, we measured 3–4 apical and 3–4 lateral epithelial regions that were averaged to give apical and lateral TG2 staining intensity per image. For visualization of TG2 protein staining, brightness and contrast was adjusted equally.

### Western blot analysis

Epithelial cell lysate samples were diluted 3:1 with 4x Laemmli buffer containing β-mercaptoethanol and boiled for 5 min. Lysate proteins were separated with SDS-PAGE using 4–20% TGX gels (Bio-Rad, CA, US), before blotting on nitrocellulose membranes using semi-dry transfer. The membranes were blocked in 5% milk/TBS-T (w/v). TG2 was detected using a custom made polyclonal rabbit anti-human TG2 antibody (1:20000 (w/v) dilution). Visualization was done using a polyclonal goat anti-rabbit Ig-HRP antibody (Southern Biotech, AL, US) and Supersignal West Pico PLUS chemiluminescent substrate. TG2 standard curves were generated using recombinant human TG2 expressed in Sf9+ insect cells [12]. Quantitative data from western blots were obtained using (ImageJ version 1.52a, National Institutes of Health, US). Detection of TG2 activity was done as in [9]. Briefly, FT lysed UCeD EDTA- fraction was incubated with or without a biotinylated ω-gliadin 34-mer (biotin-QPQQPFPQQPQQPQQPFPQPQQPFPWQPQQPFPQ, GL Biochem, China), CaCl_2_ and a TG2 inhibitor (DP3-3). After SDS-PAGE separation and transfer onto nitrocellulose membrane, the membrane was blocked in 3% bovine serum albumin/TBS-T (w/v). Gluten incorporation was visualised using streptavidin-HRP.

## Results

### TG2 is expressed in the apical epithelium of the small intestine

**Fig 1:**
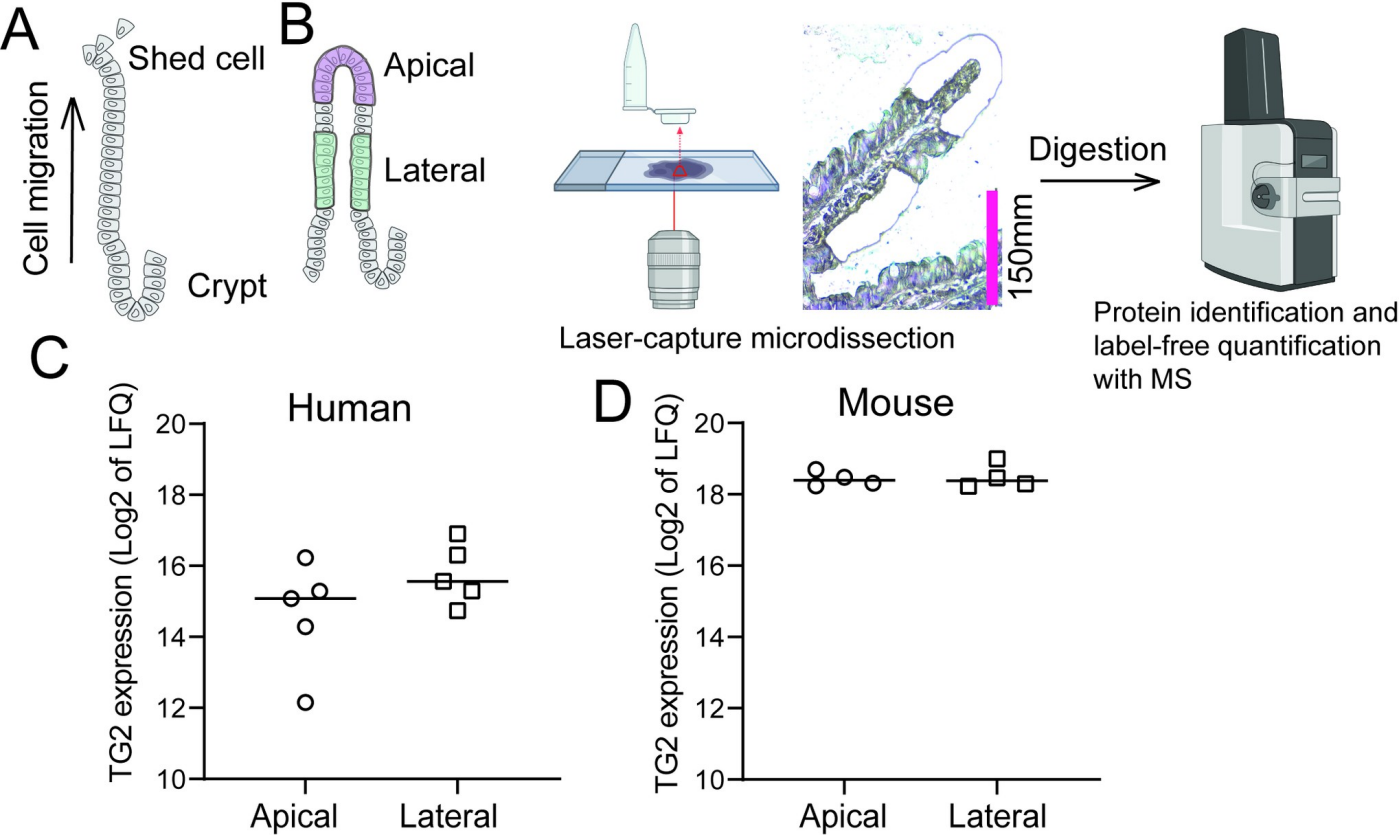
Spatially resolved analysis of TG2 protein in intestinal epithelium. A) Schematic presentation of the small intestinal epithelium. Intestinal stem cells differentiate and migrate from the crypt along the villous axis to the top where they are shed into the gut lumen. B) Workflow for LCM isolation of spatial epithelial compartments for MS based proteome analysis C-D) LFQ normalised TG2 expression in apical and lateral epithelial compartment isolated from FFPE tissue sections from human (C) and mouse (D) small intestine. (Human intestine; n = 5 apical and n = 5 lateral samples collected from three non-CeD CTR biopsies. Mouse intestine: n = 4 apical and 4 = lateral samples collected from duodenal biopsies of two mice).

We previously demonstrated that TG2 enzyme is expressed and catalytically active in mouse small intestinal epithelial cells isolated by mechanical disruption [9]. Only epithelial cells close to the villus tip are shed into the gut lumen (Fig 1A). Many proteins display a spatial expression gradient along the crypt villus axis [18, 19]. To act as a gut lumen enzyme, a prerequisite is that TG2 is expressed in villus tip (apical) epithelial cells, and it will be of interest to demonstrate this both in mouse and human small intestine. To this end we used LCM and MS based proteome analysis to assess TG2 expression in epithelial cells segmented into two spatially distinct compartments (apical and lateral) (Fig 1B) [20]. Comparing protein expression of apical regions (representing the location from which cells are shed) to that of lateral regions we found that TG2 was comparably expressed in both compartments both in human and mouse small intestine (Fig 1C and 1D). Thus, TG2 protein is constitutively expressed in the epithelial cell layer region from which cells are continuously shed into the gut lumen both in mouse and human.

### TG2 protein expression is increased in apical epithelium in coeliac disease

**Fig 2:**
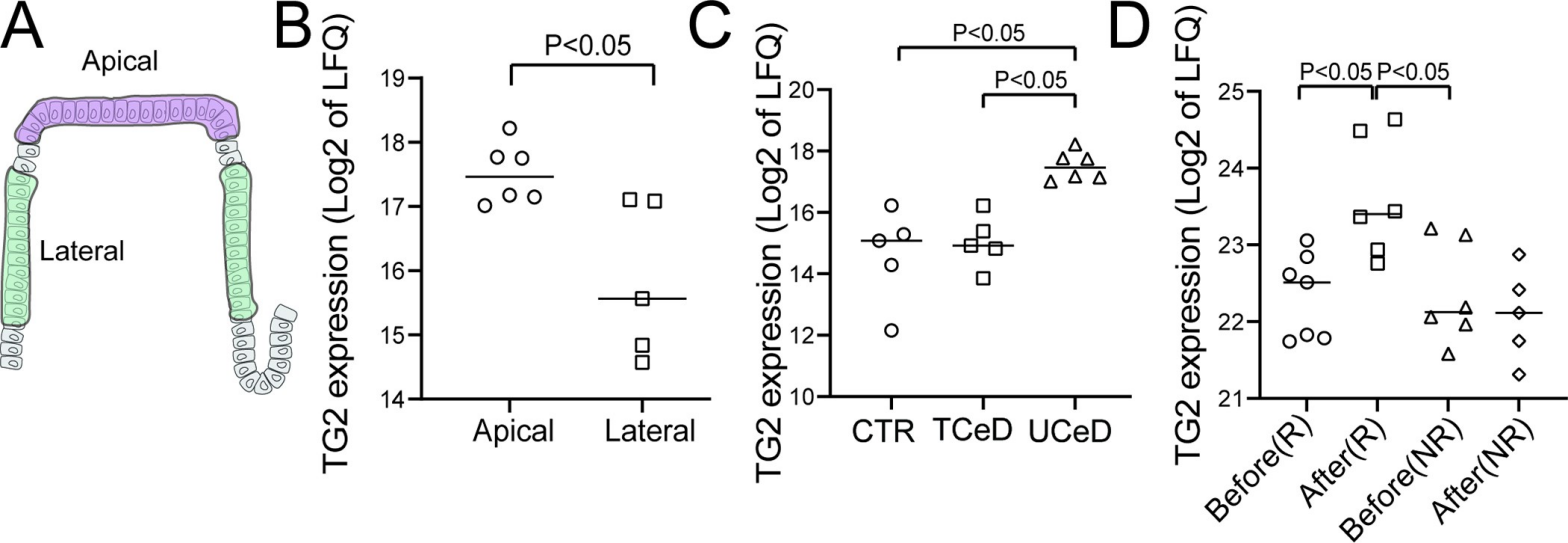
Expression of TG2 in epithelial cell layer in CeD. A) Schematic presentation of spatial epithelial compartments as isolated by LCM from untreated CeD (UCeD) patient biopsy tissue sections. B) Comparison of LFQ normalised TG2 expression in apical versus lateral epithelial cell layer compartments in UCeD intestine (n = 6 apical samples, n = 5 lateral samples from three UCeD biopsies). C) Comparison of LFQ normalised TG2 expression in apical epithelial cell layer compartment of non-CeD control (CTR, n = 5 samples from three CTR subjects), treated CeD (TCeD, n = 5 samples from three TCeD subjects) and UCeD (n = 6 samples from three UCeD subjects) intestine. D) Comparison of LFQ normalised TG2 expression in LCM isolated total epithelial cell layer of biopsies collected from 19 TCeD patients before (B) and after (A) 14-day gluten challenge. Patients were grouped as “responders” (R) and “non-responders” (NR) to gluten challenge based on mucosal proteome changes [14]. Each point represents mean expression value for LCM samples collected from one biopsy. Statistics were performed using Mann-Whitney test with significance level set at 0.05.

In CeD, the adaptive immune response to gluten causes inflammation that leads to dramatic changes in intestinal morphology with crypt elongation, flattening of the villi and loss of absorptive surface area [21]. These changes likely affect the spatial expression pattern of many epithelial proteins. To address if and how gluten-induced inflammation and remodelling affect TG2 expression in the epithelial cell compartment, we compared protein expression of apical and lateral epithelial regions from tissue section of biopsies from UCeD subjects (Fig 2A). In contrast to the healthy intestine, TG2 expression in UCeD subjects was significantly increased in the apical epithelial cells compared to lateral epithelial cells (Fig 2B). We next compared apical epithelial TG2 expression in UCeD, TCeD and CTR intestine. To better relate expression of TG2 per number of epithelial cells, we normalised the TG2 signal to the signal for villin-1, as this protein is expressed only in epithelial cells independently of their maturation status [22]. Both LFQ normalised and villin-1 normalised apical TG2 protein expression was significantly increased in UCeD compared to TCeD and CTR (Fig 2C and S1A Fig). Altered protein expression in UCeD may reflect long-term chronic inflammation. To determine whether the increase in epithelial TG2 represent an early or late feature during intestinal remodelling in CeD, we determined LFQ normalised as well as villin-1 normalised epithelial TG2 expression in a previously published dataset from TCeD patients subjected to a 2-week oral gluten challenge [14, 23]. We found that the patients who developed intestinal inflammation by day 14 after challenge (responders; R), had a significant increase in epithelial TG2 protein expression (Fig 2D and S1B Fig). These data suggest that the increase in epithelial cell layer TG2 expression occurs early during gluten induced remodelling in CeD.

**Fig 3:**
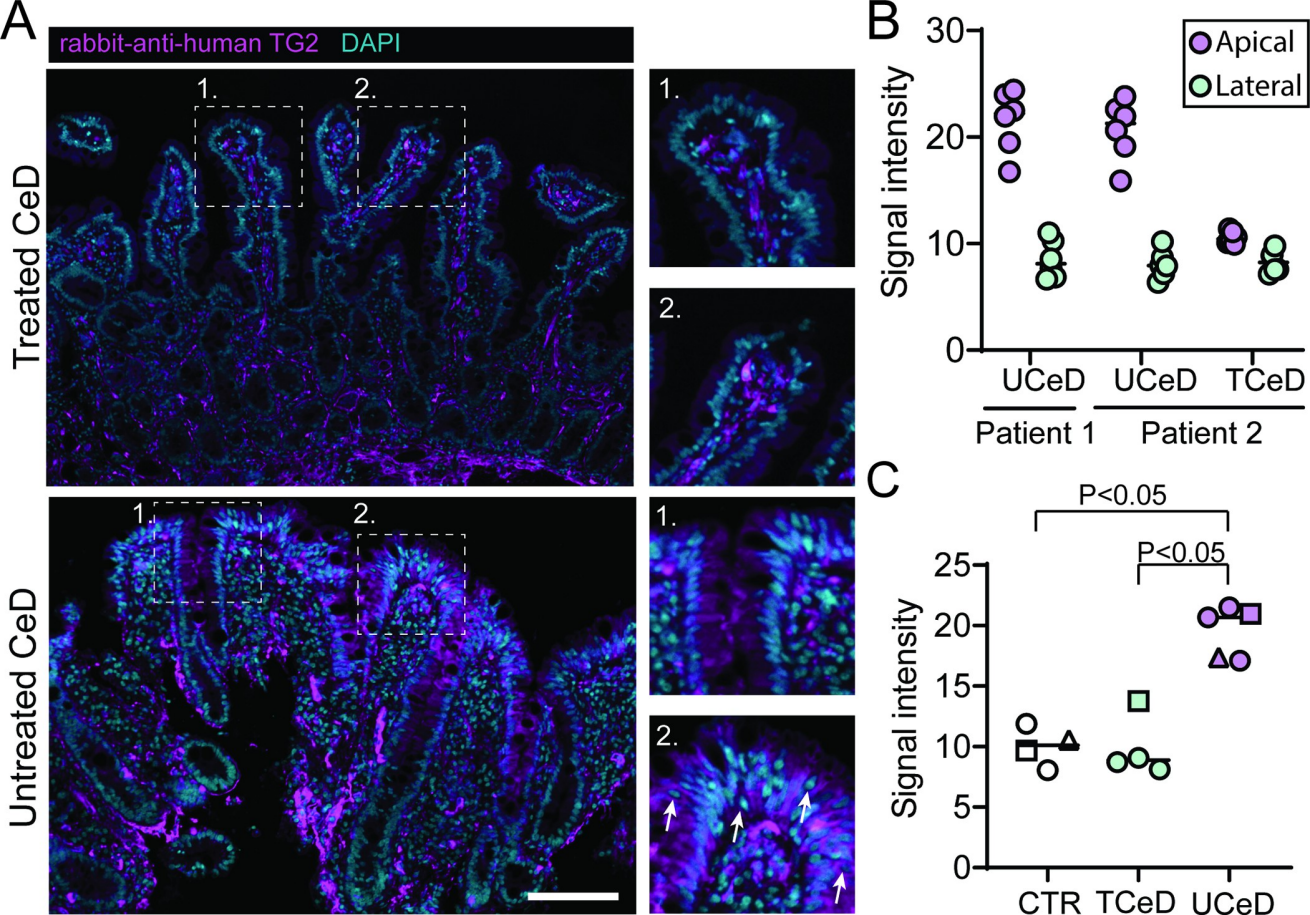
TG2 protein expression measured by immunofluorescence staining of tissue sections. A) Representative immunofluorescence staining for TG2 protein in FFPE section from UCeD and TCeD patient biopsies. Arrows in UCeD inset 2 indicate intraepithelial lymphocytes which are negative for TG2 staining. Scale bar; 100μm. B) Signal quantification in apical versus basal epithelial regions from three biopsies as described in Materials and Methods. Each point represents one annotated region C) Comparison of mean apical TG2 protein signal intensity from images of biopsies from CTR (n = 3), TCeD (n = 2) and UCeD (n = 3) subjects. Each point represents values from one image and each symbol represents one biopsy. Statistical comparison was performed using Mann-Whitney test with significance level set at 0.05.

The increased apical epithelial TG2 expression in UCeD was confirmed by antibody staining of tissue sections for TG2 protein (Fig 3A) including a quantification of the fluorescence signal (Fig 3B and 3C). Intraepithelial lymphocytes (IEL) are abundant in UCeD and will be co-isolated with epithelial cells upon LCM capture. However, as TG2 is not expressed in T cells [24], IELs should not contribute to the TG2 protein signal. Antibody staining confirmed that TG2 protein is expressed in epithelial cells and not intraepithelial lymphocytes (Fig 3A). In summary, we show by two different approaches that in UCeD, there is increased TG2 protein expression in the apical epithelial cell compartment.

### Increased TG2 expression and high epithelial cell turnover facilitate abundance of TG2 in gut lumen in UCeD

**Fig 4:**
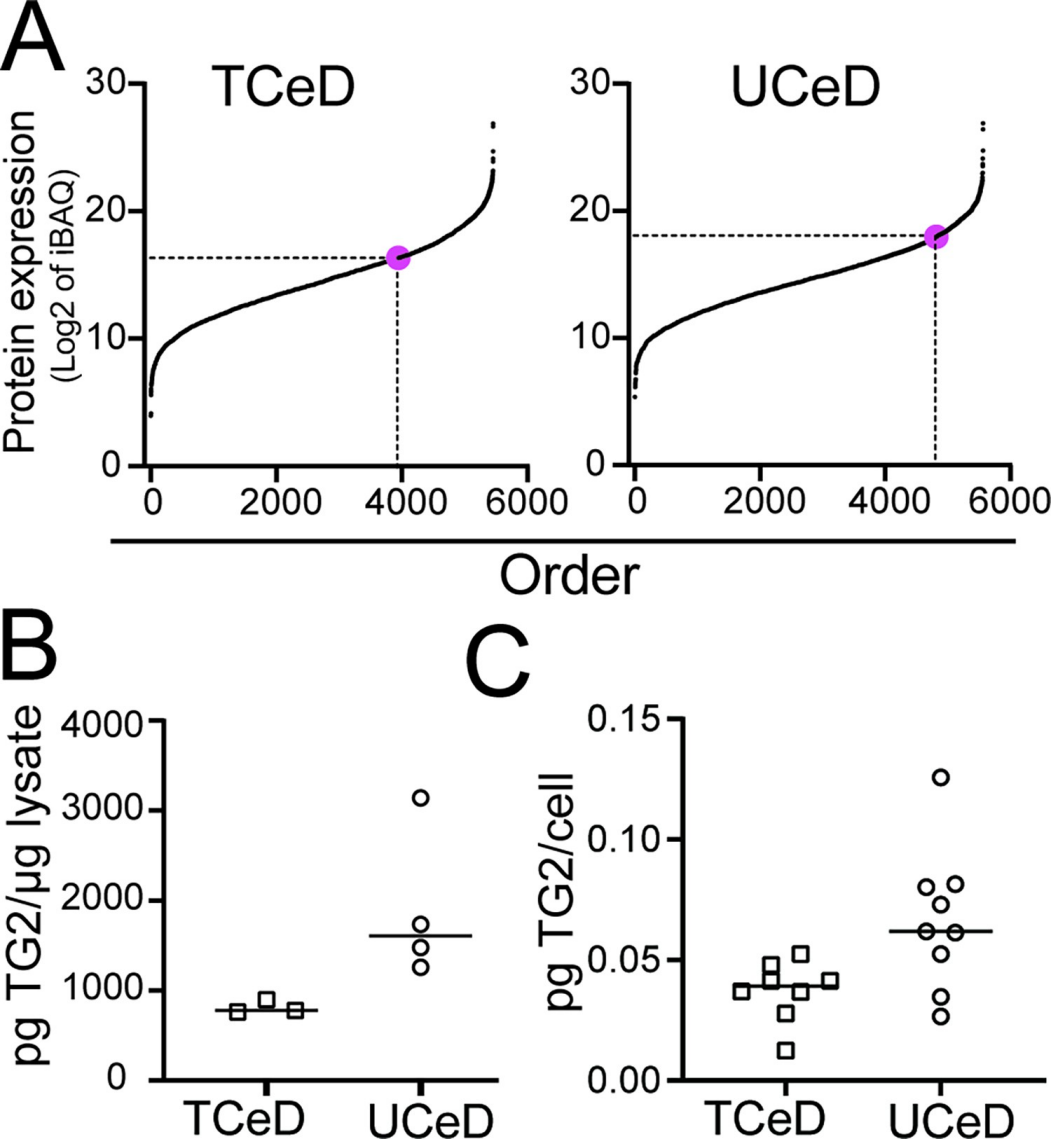
Comparison of TG2 expression in EDTA fractionated epithelial cells collected from UCeD and TCeD biopsies. A) Representative rank plot of proteins quantified by MS analysis of EDTA fractionated epithelial cells lysed by use of detergent-containing RIPA buffer. TG2 is indicated as pink dots. B) Quantification of TG2 per μg cell lysate by use of Western blot analysis. The plot depicts average values of two independent experiments. C) Amount of TG2 per epithelial cell was calculated from MS proteomics data from RIPA lysed EDTA fractionated epithelial cells using the proteomics ruler approach.

LCM isolation of tissue compartments from formalin fixed intestine retains spatial information, but may suffer from limited protein identification depth due to protein cross linking and small sample amount. To ensure comprehensive profiling of the epithelial proteome, we analysed EDTA fractionated epithelial cell cells from fresh duodenal biopsies of TCeD and UCeD patients. From the EDTA fractionated epithelial samples, we identified on average 4850 proteins (n = 34) compared to 3396 proteins (n = 25) from our LCM isolated samples. In EDTA fractions of UCeD biopsies, TG2 ranked on average as the 22% most highly expressed protein (range 13–37%) compared to TCeD where TG2 was the 33% most highly expressed protein (range 24–52%) (Fig 4A, S2 Fig). From Western blot analysis of EDTA fraction lysate, we observed a 2-fold increase in TG2 expression in UCeD biopsies compared to TCeD biopsies (Fig 4B, S3A and S3B Fig). From our proteomics data we estimated TG2 protein expression per cell by normalising against histone protein expression (proteomic ruler approach) [25]. From this estimate we confirm an approximate 2-fold increase in TG2 per cell in UCeD compared to TCeD (0.066 pg/cell vs 0.037 pg/cell) (Fig 4C). Thus, in UCeD, twice as much TG2 protein can be released from every epithelial cell shed into the gut lumen.

The number of shed cells is determined by epithelial cell turnover rate. While the turnover rate in the healthy small intestine is 4–5 days, this rate is dramatically increased in active CeD, estimated to be as short at 24 h [26]. In the healthy small intestine, approximately 2±1 x 10^10^ cells are shed into the gut lumen every 24 hours [27] which would equal ~0.83 x 10^9^ cells/hour. A four-fold increase in epithelial turnover rate in UCeD [26] would therefore equal ~3.3x10^9^ cells/hour. A combination of increased epithelial TG2 expression and increased epithelial cell turnover rate potentially releases eight times more TG2 protein into the gut lumen during active CeD. Based on our quantitation of TG2 expression in epithelial cells, a rough estimate would then suggest that potentially ~200 μg TG2 can be shed into the gut lumen every hour in UCeD patients. Of note, this calculation does not take into account the loss of apoptotic epithelial cells that do not reach the lumen due to phagocytosis by macrophages or dendritic cells in the lamina propria [28].

### TG2 is enzymatically active and readily released from human epithelial cells

**Fig 5:**
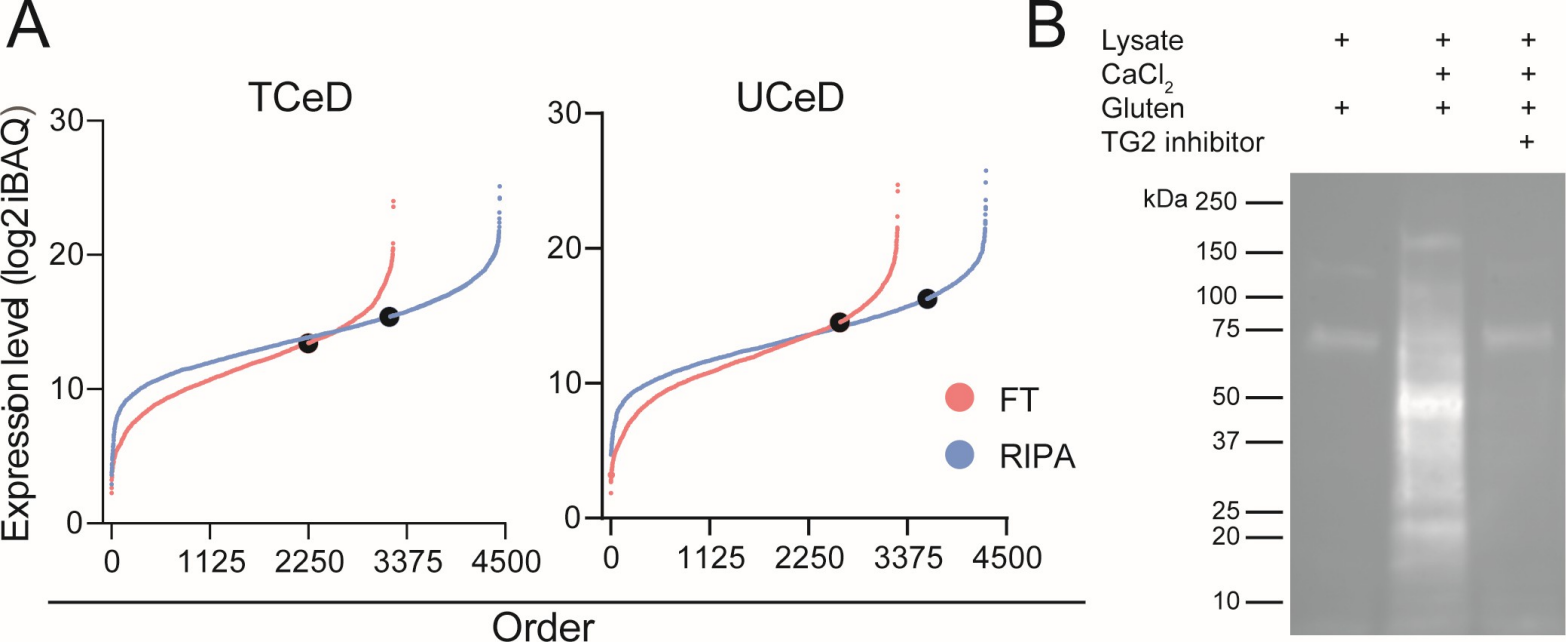
Release of active TG2 enzyme by epithelial cell rupture. A) Rank plot comparison of detected proteins released from EDTA fractionated epithelial cells lysed by use of detergent-containing RIPA buffer or freeze-thaw (FT) lysis. TG2 is indicated as black dots B) Detection of TG2 enzymatic activity by Western blot analysis. Briefly, FT lysate of EDTA fractionated epithelial cells was incubated with a biotinylated 34-mer gluten peptide (gluten) and calcium, and TG2 activity was measured from crosslinking of gluten peptide to cell lysate proteins. Sample where TG2 inhibitor was added serves as negative control.

To exert its function as a gluten catalyst in the gut lumen, TG2 must be released as active enzyme from the cytosol of shed epithelial cells. To address whether TG2 is readily released upon cell rupture, we compared lysate composition and relative TG2 protein expression following detergent-based lysis and detergent-free freeze-thaw (FT) lysis of EDTA fractions from UCeD and TCeD biopsies. As expected, cell lysis by use detergent released more proteins compared to FT lysis, but TG2 was readily released by both approaches (Fig 5A). Also, in FT lysate we found that the amount of TG2 was higher in UCeD samples than in TCeD samples (S4 Fig). The TG2 protein that was released upon FT lysis was enzymatically active as shown by calcium-dependent incorporation of biotinylated gluten peptide into cell lysate proteins (Fig 5B). In summary, we demonstrate that enzymatically active TG2 is readily released upon free-thaw rupture of human small intestinal epithelial cells.

## Discussion

Crosstalk between gluten-specific T cells and TG2-specific B cells is increasingly recognised as a central step in the pathogenesis of CeD [4, 29]. In this context, a long-standing unanswered question is where TG2:gluten complexes can form to be taken up by TG2-specific B cells. Formation of such complexes requires the presence of active enzyme and high concentrations of gluten substrate. We recently proposed that these requirements could be met in the gut lumen where dietary gluten peptides at high concentration encounter TG2 released from shed epithelial cells. Subsequently, such TG2:gluten complexes can be taken up by TG2-specific B cells localised in organised gut associated lymphoid structures. A prerequisite for this model is that the epithelial cells that are shed into the gut lumen express reasonable amounts of enzymatically active TG2 enzyme. It will be important to establish this factor in humans. Here, we demonstrate using human duodenal biopsy material that epithelial cells in the villous tip constitutively express TG2, and that enzymatically active TG2 is readily released from such cells upon cell rupture. In UCeD there is a two-fold increase in epithelial TG2 expression. Combined with a dramatic increase in cell turnover rate, this should give release into the gut lumen of more than hundred microgram TG2 per hour. Relevant to these observations, a clinical trial with oral administration of the small molecule, irreversible TG2 inhibitor ZED1227 30 min prior to consumption of gluten containing biscuits, demonstrated that the TG2-inhibitor prevented gluten-induced gut remodelling and relapse of disease during a 6-week gluten challenge [30]. ZED1227 has poor systemic uptake [31]. In light of the trial design and this fact, the convincing clinical effect of this drug may well reflect inhibition of TG2 in the gut lumen.

A recent transcriptomic study of mouse intestinal cells revealed that many protein transcripts, including TG2, have higher expression in the shed epithelial cells compared to the tissue epithelial cells [32]. The authors concluded that their observation suggests potential functions of shed cells in the intestinal lumen, which is indeed aligned with our proposed model of a function of luminal TG2 in CeD [9]. The same transcriptomic study also demonstrated that gut epithelial from heathy mice are intact and viable in the small intestinal lumen for at least an hour after being shed from the villous tip—a finding which resonates well with observations of viable epithelial cells sampled from the gut lumen of jejunum in healthy humans [10].

In 1985, several years before TG2 was identified as autoantigen in CeD [33], and as the enzyme involved in the creation of deamidated gluten T-cell epitopes [2, 3], Bruce et al. reported that TG2 has preference for gliadin as substrate and moreover, that the catalytic transglutaminase activity is increased in jejunal biopsies of patients with UCeD compared to controls subjects and patients with inflammatory bowel disease [34]. The increased catalytic activity observed in UCeD in this pioneering study could at least in part be explained by increased epithelial cell expression of TG2.

In this study we show that rather gentle cell disruption is sufficient to release high amounts of enzymatically active TG2 from isolated human intestinal epithelial cells. However, this study does not address whether TG2 will be released into gut lumen from shed enterocytes. The fact that the majority of enterocytes captured in the jejunal lumen in healthy human volunteers over a period of 2 hours followed by immune staining are indeed viable [10], suggests that apoptosis/anoikis-induced non-release of cytosolic content in enterocytes by autophagy is not a dominating mechanism in the gut. To address the issue of release of TG2 by shed enterocytes, *in vivo* studies in mice or humans will be required.

Epithelial cell turnover rate will influence the amount of epithelial cells that end up in the gut lumen. In CeD, crypt hyperplasia and tissue remodelling is accompanied by a dramatic increase in turnover rate as measured from radioactive thymidine incorporation in *ex vivo* biopsy explant cultures [26]. We observed increased epithelial TG2 expression in active CeD, and this increase appears to occur early after gluten challenge. This may suggest that epithelial cell derived TG2 in the gut lumen will serve as an important amplification step during the early phases of the recall response towards gluten. Transient conditions that increase epithelial cell turnover rate, such as infections, may have a similar effect. An increase in apoptosis, as has been reported in active CeD [35, 36] can also affect how much TG2 reaches the gut lumen. Our demonstration of increased TG2 expression in apical epithelial cells which must have avoided cell death during active CeD suggests that a substantial amount of TG2 should reach the gut lumen in active disease.

TG2 can form both stable and transient covalent complexes with gluten peptides. We recently showed that TG2-specific B cells predominantly take up transient complexes where the gluten peptide is bound to the active site cysteine of TG2 [12]. Due to the relative short half-life of such complexes, both TG2 and gluten must meet in close proximity to the TG2-specific B cells. In mice, B cells located in the sub-epithelial dome of Peyer’s patches can take up protein antigen complexes from the gut lumen [37]. Presence of TG2-specific B cells and gluten-specific T cells in Peyer’s patches has not been studied in humans as such tissue is difficult to study. Uptake of luminal TG2 gluten complexes can be modelled in genetically engineered mice that express the CeD relevant B-cell receptors [4] and T-cell receptors [38]. Hopefully, future studies of such mice can shed further light on the role of luminal TG2 in CeD.

Taken together, we demonstrate that two important prerequisites for TG2 to serve as a gut lumen enzyme interacting with gluten and as B-cell antigen in CeD are fulfilled. We find that TG2 is expressed in human epithelial cells, particularly at the villous tip, implying that the enzyme is shed into the gut lumen at appreciable levels, and we demonstrate that TG2 of human duodenal epithelial cells is catalytically active. A scenario with increased luminal TG2 activity in active CeD is suggested based on increased enterocyte TG2 expression and augmented enterocyte turnover.

## Supporting information

S1 Table CeD patient biopsies. The table provides information about human duodenal biopsies used in this study.
(DOCX)

S1 Fig Villin-1 normalised TG2 expression values. (A) Comparison of TG2 expression value (iBAQ) normalized to Villin-1 expression (iBAQ) from the apical cell region in non-CeD CTRs (n = 5 samples from three CTR subjects), TCeD (n = 5 samples from three TCeD subjects) and UCeD patients (n = 6 samples from three UCeD subjects). (B) Comparison of Villin-1 normalised TG2 expression in CeD patients before and after a 14-day gluten challenge with responders (R) and non-responders (NR) Each point represents mean expression value for LCM samples collected from one biopsy. Statistics were performed using Mann-Whitney test with significance level set at 0.05.
(TIF)

S2 Fig Villin-1 normalised TG2 expression from RIPA-lysed EDTA fraction samples. Each point represents the mean value of technical duplicates from one patient sample. Statistics were performed using Mann-Whitney test with significance level set at 0.05.
(TIF)

S3 Fig Representative Western blot for TG2 quantification. RIPA-lysed EDTA-fraction of duodenal biopsies from (A) TCeD patients and (B) UCeD patients. For both blots, recombinant human TG2 (rTG2) in TBS was used for making the standard curve. TG2 was visualised using a polyclonal rabbit anti-TG2 antibody.
(TIF)

S4 Fig Villin-1 normalised TG2 expression from FT-lysed EDTA fraction samples. Each point represents one sample from one patient.
(TIF)

S1 Data (ZIP)

S1 Raw images (PDF)

## Abbreviations

CeD: coeliac disease
CTR: control
FFPE: formalin fixed paraffin embedded
FT: freeze-thaw
IEL: intraepithelial lymphocytes
LC: liquid chromatography
LCM: laser capture microdissection
LFQ: label-free quantification
MPA: mobile phase A
MPB: mobile phase B
MS: mass spectrometry
TCeD: treated coeliac disease
TG2: transglutaminase 2
UCeD: untreated coeliac disease
