# Supplementary material for: Expression of transglutaminase 2 in human gut epithelial cells: Implications for coeliac disease
Source: PLoS One. 2023 Jun 27;18(6):e0287662. doi: 10.1371/journal.pone.0287662 (PMC10298751; doi:10.1371/journal.pone.0287662)
Supplement: S1 Table — The table provides information about human duodenal biopsies used in this study. (DOCX) [file pone.0287662.s001.docx]

| Pasient number | Pasient group | Use | Gender | Age | HLA | Marsh Score |
| --- | --- | --- | --- | --- | --- | --- |
| 1928 | CTR | LCM and IF | F | 24 | 2.5 | 0 |
| 1309 | CTR | LCM and IF | M | 23 | 2.5 | 0 |
| 1960 | CTR | LCM and IF | M | 87 | 8 | 0 |
| 1540 | TCeD | LCM and IF | M | 41 | 8 | 0 |
| 1591 | TCeD | LCM and IF | M | 29 | 2.5 | 0 |
| 1794 | TCeD | LCM | M | 25 | 2.5 | 0 |
| 1390 | TCeD | IF | F | 19 | 8 | 0 |
| 2407 | TCeD | EDTA | M | 28 | 2.5 | 0 |
| 2113 | TCeD | EDTA | F | 43 | 2.5 | 3A |
| 2406 | TCeD | EDTA | F | 35 | 2.5 | 0 |
| 2235 | TCeD | EDTA | F | 22 | 2.5 | 0 |
| 2431 | TCeD | EDTA | M | 42 | 2.5 | 0 |
| 2412 | TCeD | EDTA | F | 46 | 2.5 | 3A |
| 2405 | TCeD | EDTA | F | 44 | 2.5 | 0 |
| 2424 | TCeD | EDTA | M | 61 | 2.5 | 0 |
| 2163 | TCeD | EDTA | F | 44 | 2.5 | 1 |
| 1934 | TCeD | EDTA | F | 27 | 2.5 | 1 |
| 2119 | TCeD | EDTA | F | 27 | 2.5 | 0 |
| 1540 | UCeD | LCM | M | 39 | 8 | 3C |
| 1318 | UCeD | LCM and IF | M | 37 | 2.5 | 3B |
| 1794 | UCeD | LCM | M | 26 | 2.5 | 3C |
| 1591 | UCeD | IF | M | 28 | 2.5 | 3C |
| 1390 | UCeD | IF | F | 18 | 8 | 3C |
| 2508 | UCeD | EDTA | M | 29 | 2.5 | 3C |
| 2354 | UCeD | EDTA | F | 59 | n.d. | 3C |
| 2366 | UCeD | EDTA | F | 43 | 2.5 | 3C |
| 2516 | UCeD | EDTA | F | 22 | 2.5 | 3B |
| 2515 | UCeD | EDTA | M | 43 | 2.5 | 3C |
| 2527 | UCeD | EDTA | F | 26 | 2.5 | 3C |
| 2329 | UCeD | EDTA | M | 47 | 8 | 3B |
| 2387 | UCeD | EDTA | M | 48 | 2.5 | 3B |
| 2517 | UCeD | EDTA | F | 36 | 2.5 | 3C |
| 2340 | UCeD | EDTA | F | 69 | 2.5 | 3B |
| 2326 | UCeD | EDTA | F | 23 | 2.5 | 3B |
