# Supplementary figures and images for: Expression of transglutaminase 2 in human gut epithelial cells: Implications for coeliac disease
Source: PLoS One. 2023 Jun 27;18(6):e0287662. doi: 10.1371/journal.pone.0287662 (PMC10298751; doi:10.1371/journal.pone.0287662)

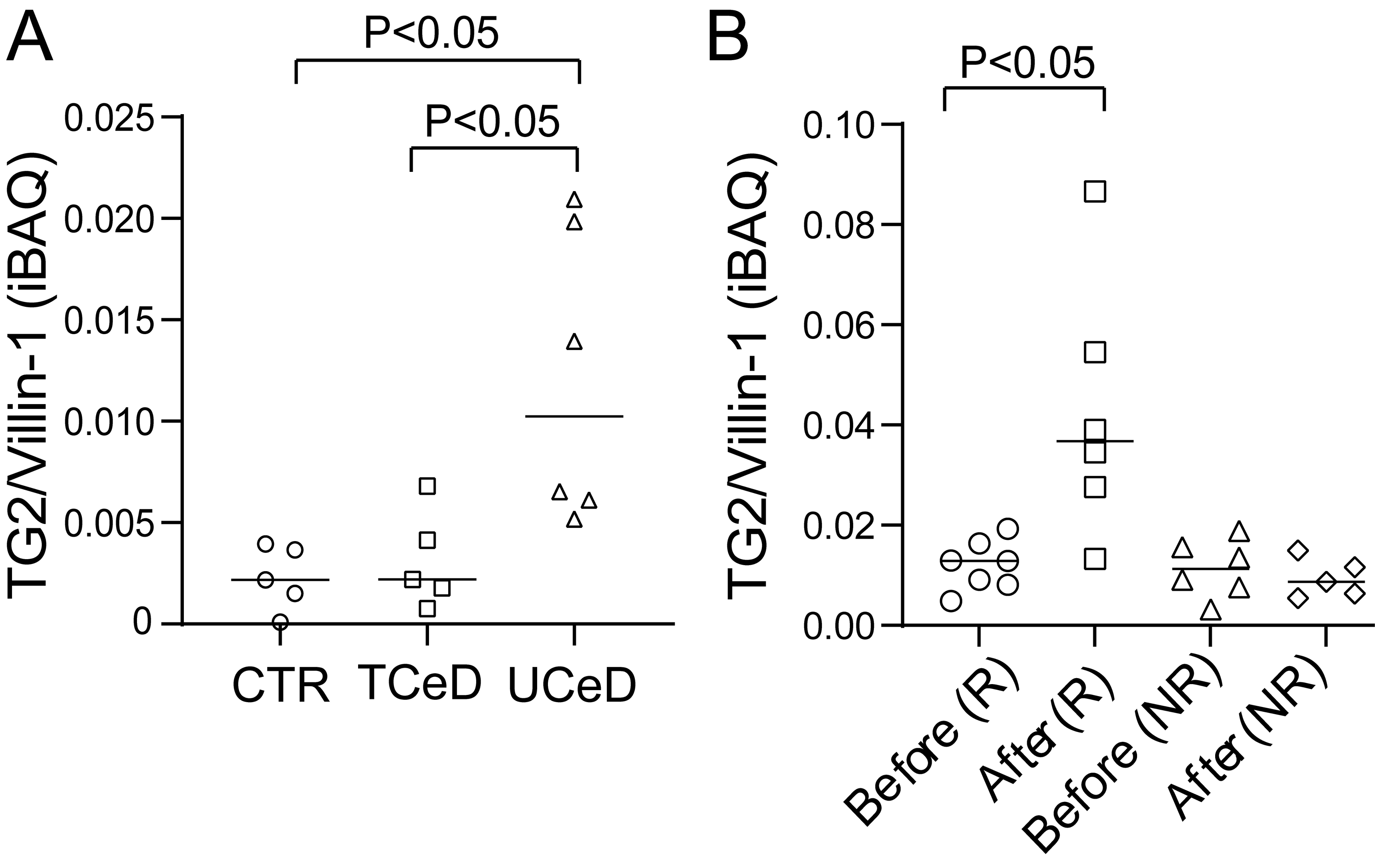

Supplement: S1 Fig — (A) Comparison of TG2 expression value (iBAQ) normalized to Villin-1 expression (iBAQ) from the apical cell region in non-CeD CTRs (n = 5 samples from three CTR subjects), TCeD (n = 5 samples from three TCeD subjects) and UCeD patients (n = 6 samples from three UCeD subjects). (B) Comparison of Villin-1 normalised TG2 expression in CeD patients before and after a 14-day gluten challenge with responders (R) and non-responders (NR) Each point represents mean expression value for LCM samples collected from one biopsy. Statistics were performed using Mann-Whitney test with significance level set at 0.05. (TIF) [file pone.0287662.s002.tif]

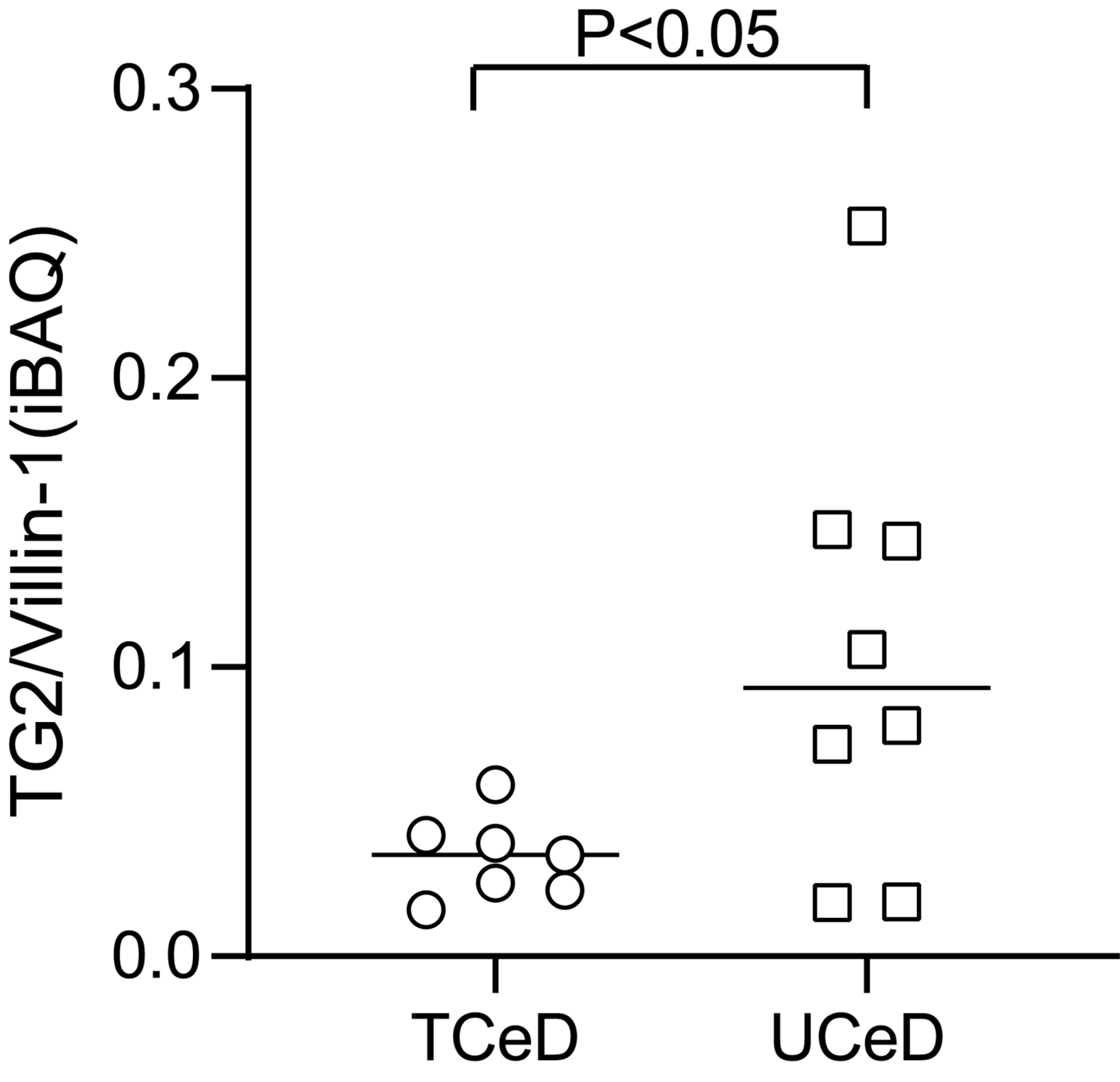

Supplement: S2 Fig — Each point represents the mean value of technical duplicates from one patient sample. Statistics were performed using Mann-Whitney test with significance level set at 0.05. (TIF) [file pone.0287662.s003.tif]

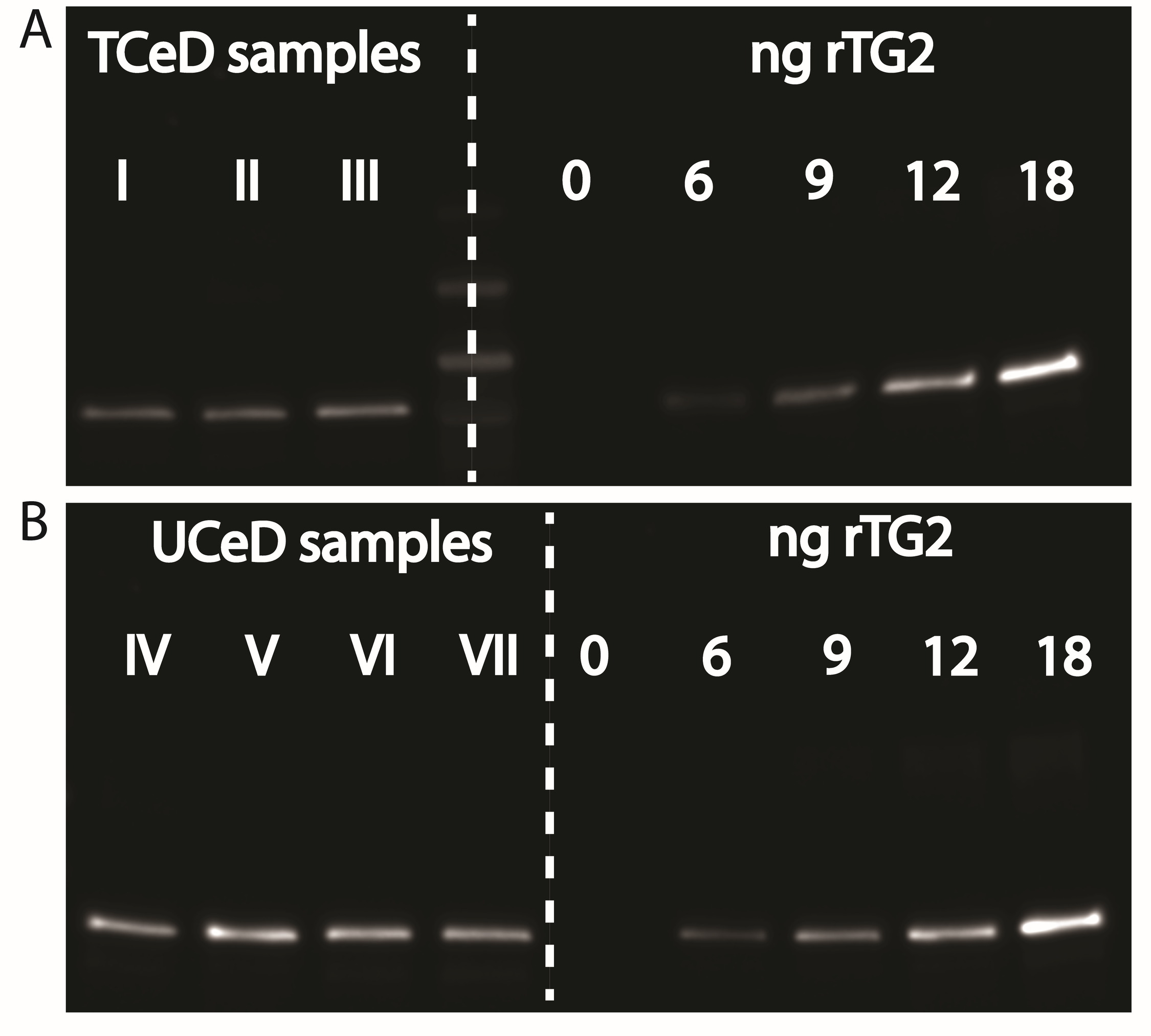

Supplement: S3 Fig — RIPA-lysed EDTA-fraction of duodenal biopsies from (A) TCeD patients and (B) UCeD patients. For both blots, recombinant human TG2 (rTG2) in TBS was used for making the standard curve. TG2 was visualised using a polyclonal rabbit anti-TG2 antibody. (TIF) [file pone.0287662.s004.tif]

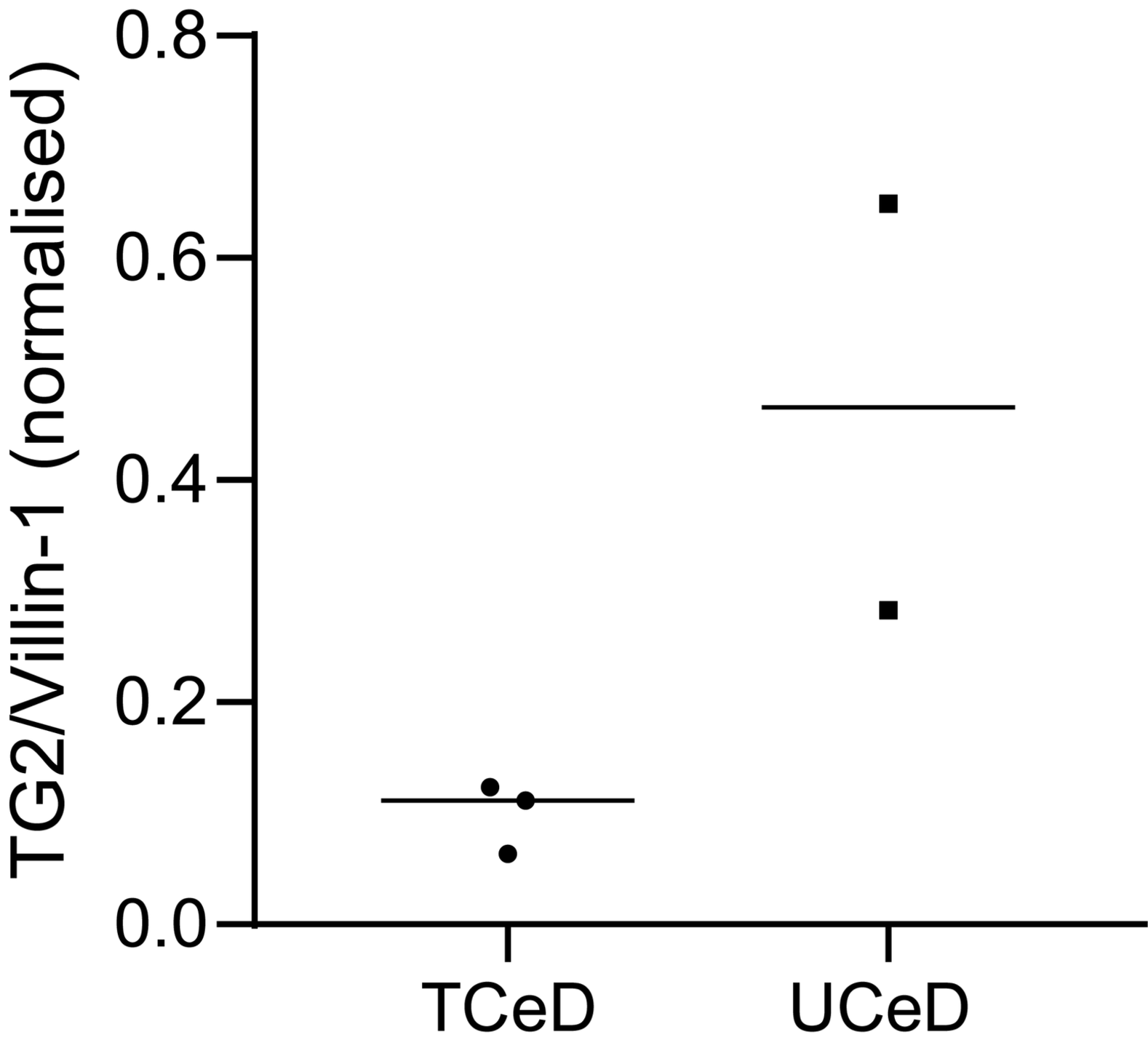

Supplement: S4 Fig — Each point represents one sample from one patient. (TIF) [file pone.0287662.s005.tif]
