# Supplementary material for: Expression of transglutaminase 2 in human gut epithelial cells: Implications for coeliac disease
Source: PLoS One. 2023 Jun 27;18(6):e0287662. doi: 10.1371/journal.pone.0287662 (PMC10298751; doi:10.1371/journal.pone.0287662)
Supplement: S1 Raw images — (PDF) [file pone.0287662.s007.pdf]

1. Original blot for Figure 4B Rep 1 and S3 (TCeD)

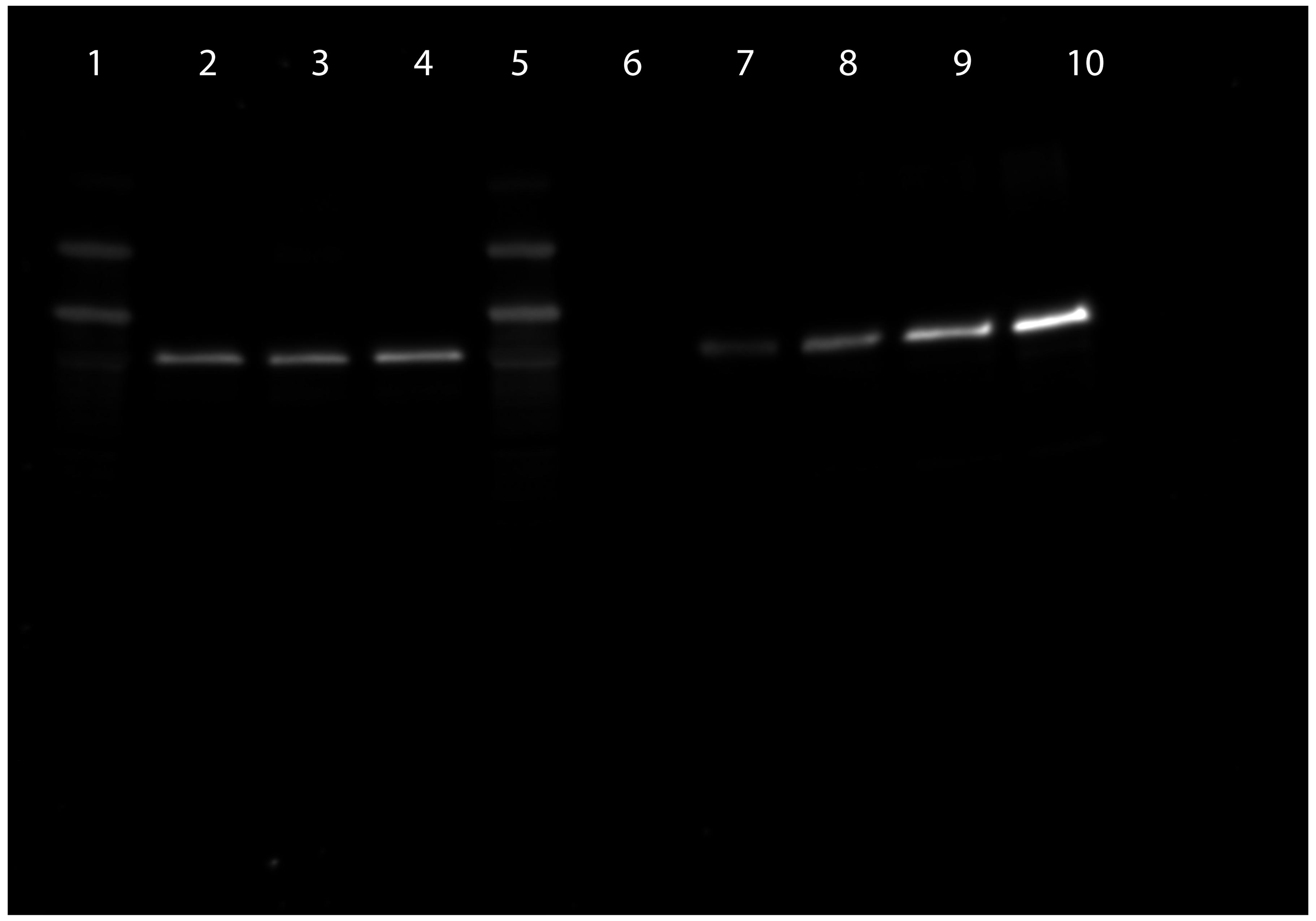

2. Original blot for Figure 4B Rep 2 (TCeD)

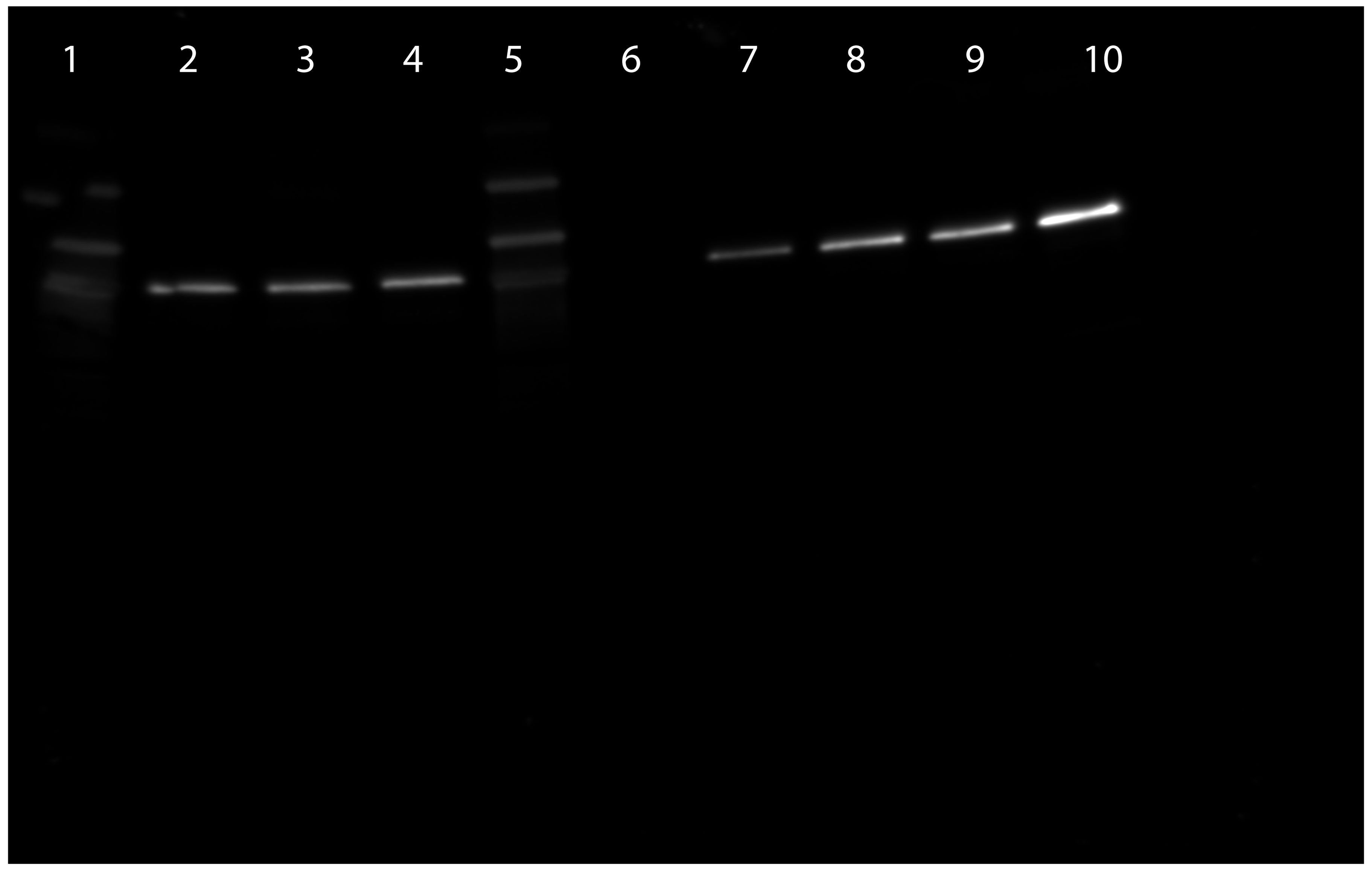

Sample order for image 1 and 2:

1) Ladder
2) TCeD pasient I
3) TCeD pasient II
4) TCeD pasient III
5) Ladder
6) TBS (used for 0 ng TG2)
7) 6 ng recombinant human TG2 (rhTG2)
8) 9 ng hTG2
9) 12 ng rhTG2
10) 18 ng rhTG2

3. Original blot for Figure 4B and S3 (UCeD) rep 1

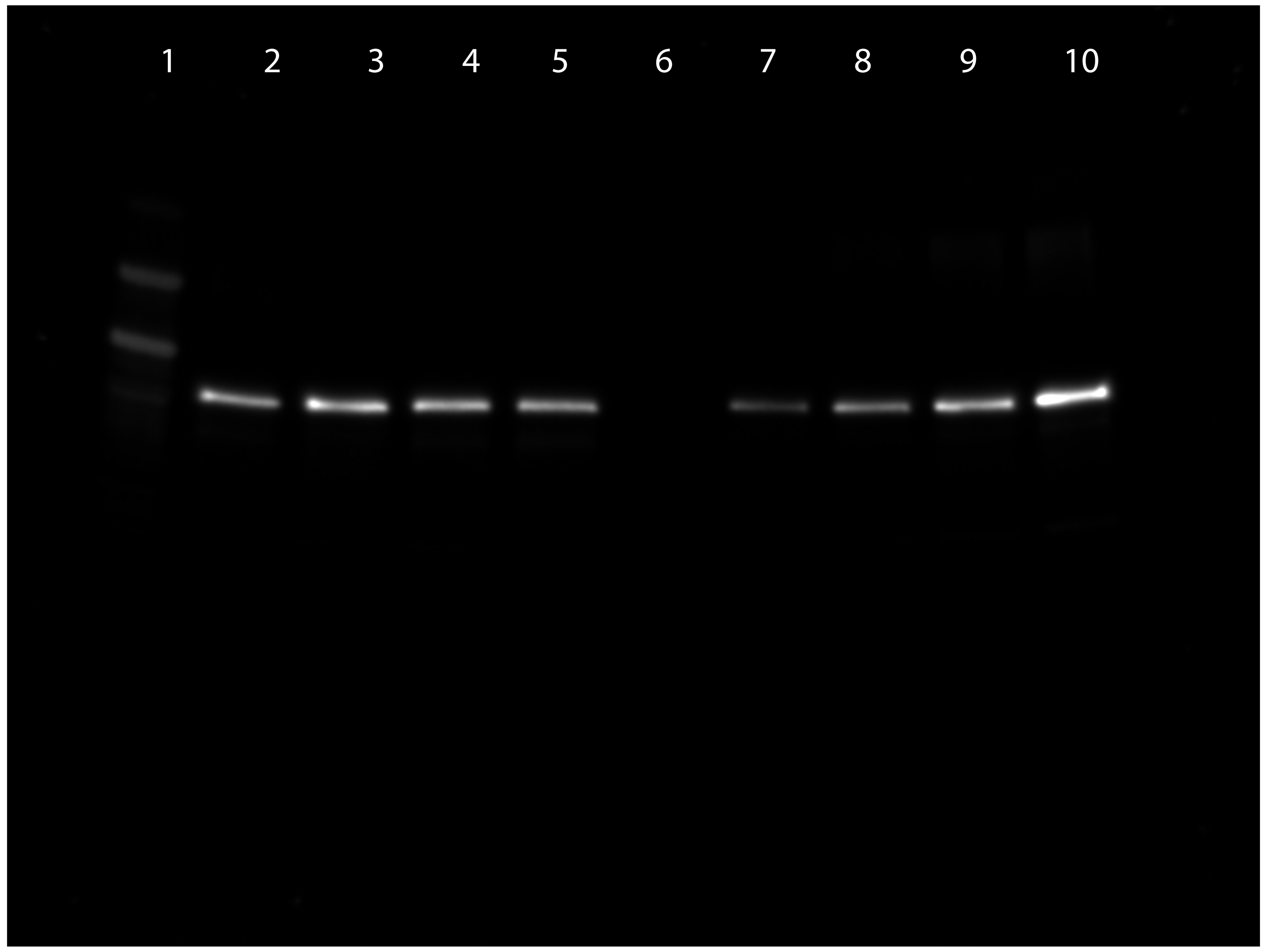

Sample order for image 3 and 4:

1) Ladder
2) TCeD pasient IV
3) TCeD pasient V
4) TCeD pasient VI
5) UCeD pasient VII
6) TBS (used for 0 ng TG2)
7) 6 ng recombinant human TG2 (rhTG2)
8) 9 ng hTG2
9) 12 ng rhTG2
10) 18 ng rhTG2

4. Original blot for Figure 4B (UCeD) rep 2

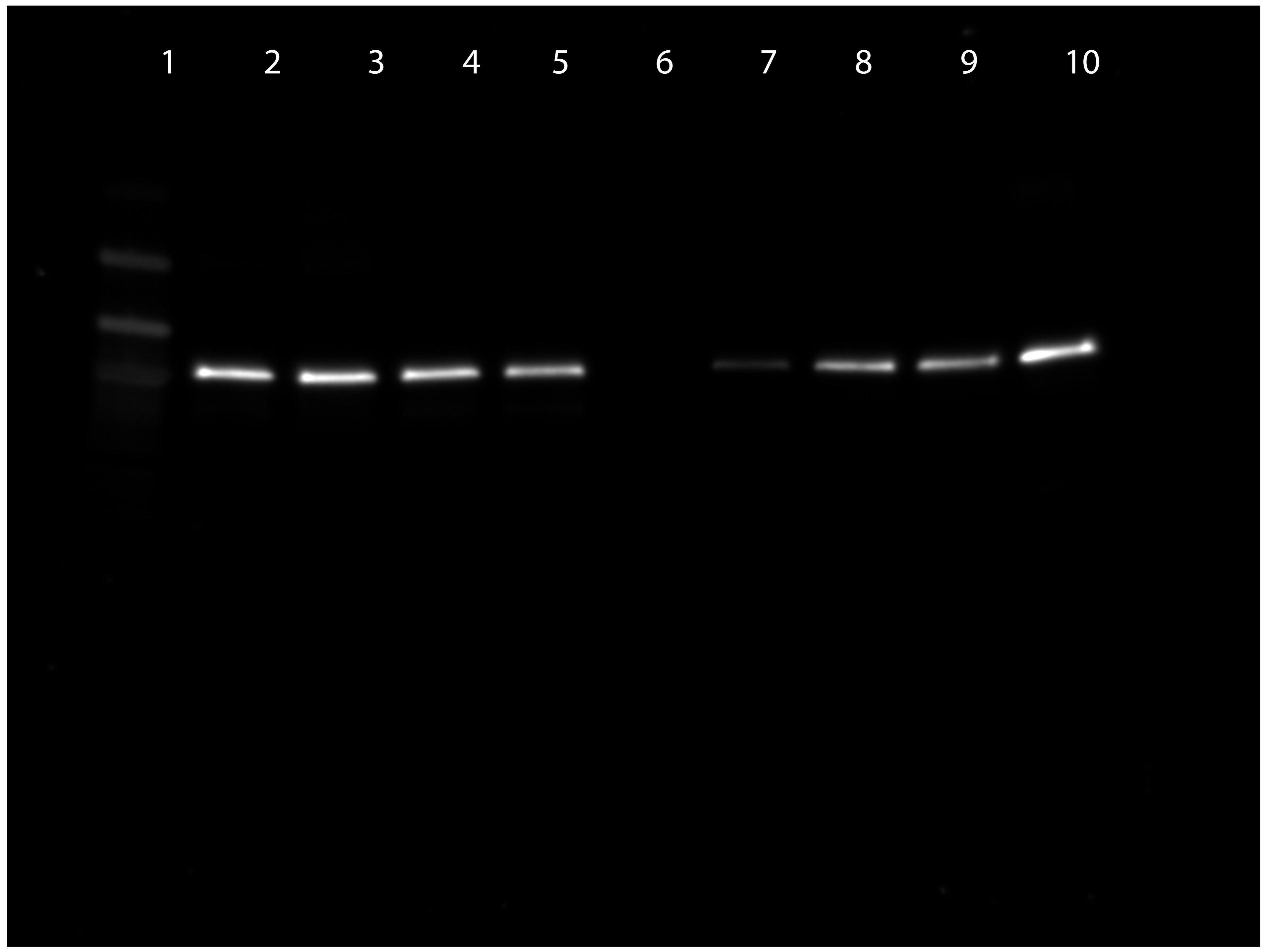

5. Original blot Figure 5

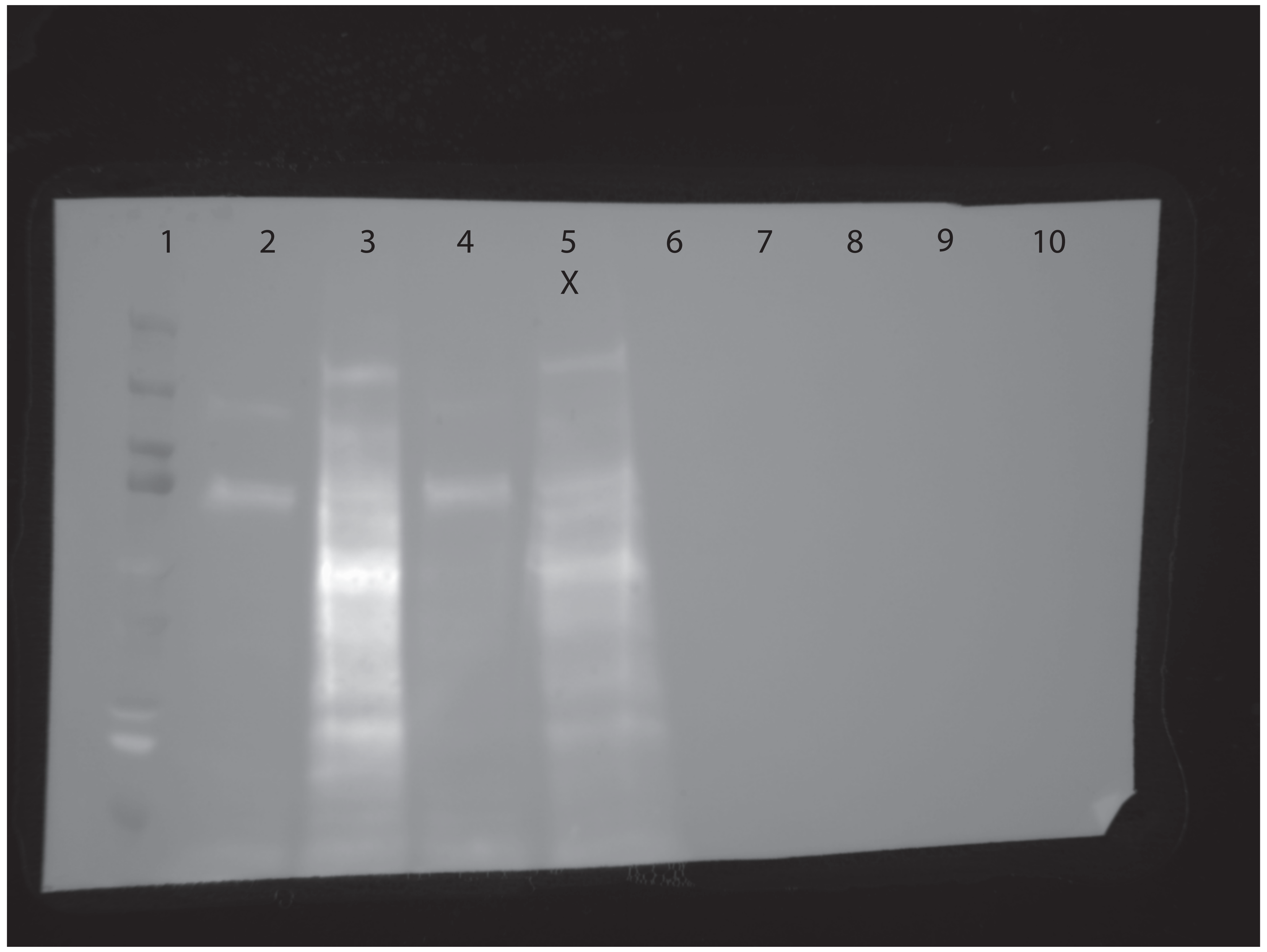

Sample order for image 3 and 4:

1) Ladder
2) UCeD lysate+ biotinylated gluten peptide
3) UCeD lysate + CaCl2 + biotinylated gluten peptide
4) UCeD lysate + CaCl2 + biotinylated gluten peptide + TG2 inhibitor
5) X
6) No sample
7) No sample
8) No sample
9) No sample
10) No sample
